# *MIR938* rs2505901 T>C polymorphism is associated with increased neuroblastoma risk in Chinese children

**DOI:** 10.1042/BSR20231223

**Published:** 2023-11-15

**Authors:** Susu Jiang, Xiuhong Sun, Xinxin Zhang, Chunlei Zhou, Haiyan Wu, Jing He, Wenhan Yang

**Affiliations:** 1 Department of Child and Adolescent Health, School of Public Health, Guangdong Pharmaceutical University, Guangzhou, Guangdong Province 510006, China; 2 Department of Pediatric Surgery, Guangzhou Institute of Pediatrics, Guangdong Provincial Key Laboratory of Research in Structural Birth Defect Disease, Guangzhou Women and Children's Medical Center, Guangzhou Medical University, Guangzhou 510623, Guangdong, China; 3 Department of Medical Ultrasonics, Guangzhou Women and Children’s Medical Center, Guangzhou Medical University, Guangzhou 510623, Guangdong, China; 4 Department of Pathology, Children’s Hospital of Nanjing Medical University, Nanjing 210008, Jiangsu, China

**Keywords:** MIR938, neuroblastoma, polymorphism, susceptibility

## Abstract

Neuroblastoma (NB) is a kind of childhood cancer that is a prevailing and deadly solid neoplasm among pediatric malignancies. The transcriptional output of *MIR938* is capable of participating in the posttranscriptional modulation of gene expression, whereby it exerts its regulatory effect by modulating both the stability and translation of target mRNAs. Previous studies showed that *MIR938* was associated with many cancers. Hence, functional genetic variants in the *MIR938* can be attributed to NB risk. We recruited 402 neuroblastoma patients and 473 controls from the Children’s Hospital of Nanjing Medical University and genotyped one *MIR938* single-nucleotide polymorphism (SNP) (rs2505901 T>C). There were significant associations between the rs2505901 T>C and NB risk [CC vs. TT: adjusted odds ratio (OR) = 1.90, 95% confidence interval (CI) = 1.02–3.55, *P*=0.045; CC vs. TT/TC: adjusted OR = 2.02, 95% CI = 1.09–3.75, *P*=0.026]. This analysis of genotypes revealed that T>C increased the risk of NB. Some borderline significant different relationships were observed in the stratified analyses: age ≤ 18 months (adjusted OR = 2.95, 95% CI = 0.92–9.51, *P*=0.070), male sex (adjusted OR = 2.19, 95% CI = 0.95–5.08, *P*=0.067), and clinical stage III+IV (adjusted OR = 2.12, 95% CI = 0.98–4.56, *P*=0.055). The present study revealed that the *MIR938* rs2505901 T>C polymorphism may be a potential risk factor for neuroblastoma in Chinese children. In the long term, conducting large and diverse sample studies from different ethnicities will indeed be crucial in determining the role of *MIR938* polymorphisms in NB risk. By including individuals from various ethnic backgrounds, researchers can account for potential genetic variations that may exist between populations.

## Introduction

Neuroblastoma (NB), an affliction that is a prevailing and deadly solid neoplasm among pediatric malignancies [1], accounts for approximately 7% of childhood cancer cases [2]. With a heterogeneous clinical range from localized or spontaneously regressing to widely metastatic disease [3], NB is strongly linked to pediatric malignancy mortality, especially in high-risk patients experiencing chemoresistant relapse, whose mortality rate is almost as high as 60% [2,4,5].

A previous study showed that NB might be associated with a few nongenetic factors, such as recreational drug use, marijuana use, hair dye use and prenatal exposure during pregnancy, but the nongenetic factor effect was very weak [6–8]. Compared with nongenetic factors, genetic factors play an important role in NB. NB cells often suffer from extensive, nonrandom genetic damage at multiple genetic loci. NB shows striking genetic heterogeneity characterized by various chromosomal/gene modifications and dysregulated expression of pivotal oncogenes that instigate tumor initiation and facilitate the advancement of the disease [1,9]. Segmental chromosome alterations such as 1p, 11q, and 14q deletions and 17q gain [5,10] and several gene alterations such as *MYCN* amplification [4,11,12], germline or somatic mutations of *ALK* [13–16] and *PHOX2B* [17,18], and rearrangements at *ATRX* [19] and at *TERT* [20] were common in NB cases, and these alterations also led to worse prognosis.

MicroRNAs (miRNAs) are a cohort of small (20–24 nucleotides) noncoding RNAs that orchestrate the posttranscriptional regulation of gene expression in multicellular organisms. Their impact is exerted through the modulation of mRNA stability and translation. Previous studies have shown that miRNA dysregulation is causal in many cases of cancer [21]. Moreover, miRNAs profiles can be surprisingly informative, revealing the developmental lineage and differentiation state of tumors [22]. *MIR938* participates in the expression of miRNAs. Current existing articles have shown that *MIR938* polymorphisms are associated with many kinds of diseases, including intestinal-type gastric cancer (intestinal-type GC) [23], colorectal cancer (CRC) [24], primary ovarian insufficiency [25], and idiopathic recurrent pregnancy loss [26].

Genome-wide association studies (GWASs) have provided evidence that common genetic variants are associated with neuroblastoma susceptibility. However, until now, no studies have revealed the relationship between *MIR938* polymorphism and neuroblastoma. Incorporating multiple testing correction in the GWAS analysis may inadvertently lead to overlooking certain potential single-nucleotide polymorphisms (SNPs) with moderate risk effects [27]. Additional gene polymorphisms need to be identified.

## Materials and methods

### Study subjects

We randomly selected 402 children with neuroblastoma and 473 children without NB from the Children’s Hospital of Nanjing Medical University (Supplementary Table S1). Neuroblastoma cases were defined by biopsy and histology and stratified by the International Neuroblastoma Staging System (INSS) [28], and children without cancer were matched by similar population characteristics. All the participants’ parents or guardians signed informed consent forms, and clinical information was collected through personal interviews [29]. This study was approved by the Children’s Hospital of Nanjing Medical University institutional review board (Approval No: 202112141-1).

### SNP selection and genotyping

The rs2505901 T>C polymorphism was chosen from our previous studies [30,31]. In addition, pertinent details regarding this selection are also available for reference. A TIANamp Blood DNA Kit was used to extract genomic DNA from peripheral blood donated by subjects. The selected *MIR938* rs12416605 polymorphism was genotyped using a standard TaqMan real-time PCR method. The primers and probes sequences manufacturer is ThermoFisher Scientific and their ID is C__16253955_10.

### Statistical analysis

We adopted the goodness-of-fit χ^2^ test to calculate Hardy–Weinberg equilibrium (HWE) in controls, the two-sided chi-square test to measure the difference in categorical variables between cases and controls and the *t*-test to measure the significance of continuous variables. Logistic regression analysis was used to test the association between candidate SNP and NB susceptibility, followed by further stratified analyses by age, sex, tumor sites, and clinical stages. All of these analyses were performed with SAS 9.4 (SAS Institute Inc., Cary, North Carolina).

## Results

### Association analysis of the rs2505901 T>C polymorphism with NB risk

There were 400 cases and 473 controls successfully genotyped. Complete information regarding the demographic profiles of all participants can be found in Supplementary Table S1, as well as in our previously published articles [29]. As shown in Table 1, the rs2505901 T>C was in accordance with HWE (HWE = 0.515). In our adjusted model, we observed a significant association between the *MIR938* rs2505901 T>C polymorphism and an increased risk to neuroblastoma [CC vs. TT: adjusted odds ratio (OR) = 1.90, 95% confidence interval (CI) = 1.02–3.55, *P*=0.044; TT/TC vs. CC: adjusted OR = 2.02, 95% CI=1.09–3.75, *P*=0.026].

**Table 1:** *MIR938* rs2505901 T>C polymorphism and neuroblastoma risk in children from Jiangsu province

Genotype	Cases (*N*=400)	Controls (*N*=473)	P*	Crude OR (95% CI)	*P*	Adjusted OR (95% CI)^†^	*P* ^†^
rs2505901 (HWE = 0.515)							
TT	259 (64.75)	299 (63.21)		1.00		1.00	
TC	113 (28.25)	157 (33.19)		0.83 (0.62–1.11)	0.217	0.83 (0.62–1.11)	0.213
CC	28 (7.00)	17 (3.59)		**1.90 (1.02–3.55)**	**0.044**	**1.90 (1.02–3.55)**	**0.045**
Additive			0.640	1.06 (0.84–1.32)	0.639	1.06 (0.84–1.32)	0.646
Dominant	141 (35.25)	174 (36.79)	0.638	0.94 (0.71–1.24)	0.638	0.93 (0.71–1.23)	0.629
TT/TC	372 (93.00)	456 (96.41)		1.00		1.00	
CC	28 (7.00)	17 (3.59)	0.023	**2.02 (1.09–3.75)**	**0.026**	**2.02 (1.09–3.75)**	**0.026**
T	631 (78.88)	755 (79.81)		1.00		1.00	
C	169 (21.13)	191 (20.19)	0.631	1.06 (0.84–1.34)	0.630	1.06 (0.84–1.34)	0.638

CI, confidence interval; HWE, Hardy–Weinberg equilibrium; OR, odds ratio.

* χ^2^ test for genotype distributions between neuroblastoma cases and cancer-free controls.

† Adjusted for age and sex.

### Stratification analysis

Furthermore, we performed additional analyses by stratifying the data according to age, sex, site of origin, and clinical stage (Table 2) to investigate the relationship between the *MIR938* rs2505901 T>C polymorphism and susceptibility to neuroblastoma.

**Table 2:** Stratified analysis for the association between *MIR938* rs2505901 T>C polymorphism and neuroblastoma susceptibility

Variable	rs2505901 (cases/controls)		OR (95% CI)	*P*	AOR (95% CI)^*^	P*
	TT/TC	CC				
Age, months						
≤18	126/135	11/4	2.95 (0.92–9.49)	0.070	**2.95 (0.92–9.51)**	**0.070**
>18	246/321	17/13	1.71 (0.81–3.58)	0.158	1.71 (0.81–3.58)	0.157
Sex						
Female	179/217	12/8	1.82 (0.73–4.55)	0.201	1.82 (0.73–4.55)	0.201
Male	193/239	16/9	2.20 (0.95–5.09)	0.065	**2.19 (0.95–5.08)**	**0.067**
Site of origin						
Adrenal gland	86/456	7/17	2.18 (0.88–5.42)	0.093	2.17 (0.87–5.39)	0.096
Retroperitoneal	155/456	11/17	1.91 (0.87–4.16)	0.105	1.91 (0.88–4.18)	0.104
Mediastinum	111/456	8/17	1.93 (0.81–4.59)	0.136	1.93 (0.81–4.59)	0.136
Others	16/456	2/17	3.35 (0.71–15.76)	0.126	3.53 (0.74–16.73)	0.113
Clinical stage						
I+II+4s	164/456	9/17	1.47 (0.64–3.37)	0.360	1.51 (0.66–3.46)	0.332
III+IV	151/456	12/17	2.13 (1.00–4.57)	0.051	**2.12 (0.98–4.56)**	**0.055**

AOR, adjusted odds ratio; CI, confidence interval; OR, odds ratio.

* Adjusted for age and sex, omitting the corresponding stratification factor.

However, no significant difference was found for the *MIR938* rs2505901 T>C polymorphism and neuroblastoma risk in the stratification analyses among different age groups (in months), sex groups, site of origin groups, and clinical stage groups. The rs2505901 T>C showed a borderline significant correlation with age, sex and clinical stage. Our study revealed that the children with age ≤18 months were more likely to carry the rs2505901 CC genotype (adjusted OR = 2.95, 95% CI = 0.92–9.51, *P*=0.070). Compared with females, males were more likely to carry the rs2505901 CC genotype (adjusted OR = 2.19, 95% CI = 0.95–5.08, *P*=0.067). Moreover, carriers of the CC genotype had an increased risk of INSS clinical stage III+IV than that of carriers of the TT/TC genotype (adjusted OR = 2.12, 95% CI = 0.98–4.56, *P*=0.055).

## Discussion

To explore the association between the *MIR938* polymorphism and neuroblastoma, we undertook a case‒control study specifically focused on Chinese children. Our study showed that the *MIR938* rs2505901 T>C polymorphism could predispose the Chinese population to neuroblastoma risk. In spite of the lack of statistical significance in the subsequent stratified studies, rs2505901 T>C showed a borderline difference correlation with age, sex and clinical stage.

*MIR938* is located on *Homo sapiens* chromosome 10, GRCh38.p14 Primary Assembly. The transcription product of *MIR938* is a kind of miRNA, which is a class of small (20–24 nt) noncoding RNAs that orchestrate the posttranscriptional regulation of gene expression in multicellular organisms [32].

Previous studies revealed that *MIR938* plays critical roles in several reproductive disorders [25,26]. *MIR938* can prevent the risk of idiopathic recurrent pregnancy loss, and its potential mechanisms to affect the female reproductive system involve the *TGF-β* signaling pathway [33]. Although the underlying mechanisms by which the *MIR938* G>A polymorphism contribute to the development of primary ovarian insufficiency have not yet been elucidated, Cho et al. showed that *MIR938* G>A polymorphism might be attributed to the regulation of POI-related target genes, mostly the gonadotropin-releasing hormone receptor [25].

On the other hand, *MIR938* plays an important role in CRC and intestinal-type GC. Landeros et al. showed that *MIR938* rs12416605 C>T was associated with intestinal-type GC risk in a Chilean population [23]. The underlying mechanism entails alteration of the initial nucleotide within the seed region of *MIR938* while leaving the secondary structure unaffected. Another study showed that *MIR938* is up-regulated in CRC tissues and cells and can promote CRC cell proliferation by inhibiting leucine-rich repeat protein phosphatase 2 [24].

Our findings indicate a significant association between the *MIR938* rs2505901 T>C polymorphism and an increased risk to neuroblastoma. However, further stratified analyses did not show significant differences among different age groups, sex groups, site of origin groups, and clinical stage groups. In the stratified analysis, we found that rs2505901 T>C showed weak correlations with age ≤18 months, male sex, and clinical stage III+IV. The rs2505901 is an upstream variant located in the intron region of the *MIR938* gene. *MIR938* can influence the regulatory pathways of cell apoptosis and survival. The GTEx portal showed that the rs2505901 TT/CT genotypes may inhibit these regulatory pathways by lowering the expression of *MIR938* compared with that in the CC genotype, and the SNP-like variants of the pre-*MIR938* gene were associated with *MIR938* biogenesis and stability. This may be a potential mechanism by which the *MIR938* rs2505901 T>C polymorphism is associated with increased neuroblastoma risk.

The present study represents the first investigation between *MIR938* rs2505901 T>C polymorphism and the risk of neuroblastoma among the Chinese pediatric population. Nevertheless, there are still some weaknesses. First, due to the limited sample size of our case‒control study among Chinese children, the statistical power of our findings might be somewhat compromised. Second, we only focused on a single polymorphism in *MIR938*; hence, the impact of *MIR938* polymorphisms may be overshadowed by other genetic factors. Further investigation of additional potentially functional polymorphisms within *MIR938* is needed. Third, polymorphisms can manifest varied genetic influences on the susceptibility to different types of cancer, owing to factors such as geographic regions and ethnic backgrounds. The lack of an observed association between the one *MIR938* polymorphism and NB may be attributed to ethnic differences. Therefore, caution is advised when extrapolating the findings to other ethnic populations. Fourth, our research group was NB patients and volunteers from the Children’s Hospital of Nanjing Medical University, not healthy volunteers, even though we matched the two groups demographically. Thus, bias may exist in the results. Fifth, our research adapted the TaqMan real-time PCR method for the genotype. The gold standard should be used for secondary analysis to ensure the first method accuracy. Lacking of validation is a limitation of the study.

In summary, our present data indicated that *MIR938* rs2505901 was significantly associated with NB risk. However, there was no marked difference in the stratification analysis. We still found a weak association between age, sex and clinical stage and NB susceptibility. Well-designed studies in large samples and diverse ethnic groups are needed to confirm our findings.

## Supplementary Material

Supplementary Table S1

## Data Availability

All the data are available upon request from the corresponding authors.

## Abbreviations

CI: confidence interval
CRC: colorectal cancer
GWAS: genome-wide association study
HWE: Hardy–Weinberg equilibrium
INSS: International Neuroblastoma Staging System
intestinal-type GC: intestinal-type gastric cancer
miRNA: microRNA
NB: neuroblastoma
OR: odds ratio
SNP: single-nucleotide polymorphism
