# Supplementary material for: MIR938 rs2505901 T>C polymorphism is associated with increased neuroblastoma risk in Chinese children
Source: Biosci Rep. 2023 Nov 15;43(11):BSR20231223. doi: 10.1042/BSR20231223 (PMC10652078; doi:10.1042/BSR20231223)
Supplement: Supplementary Table S1 [file BSR-2023-1223_supp.pdf]

Table S1. Demographic characteristics of neuroblastoma patients and cancer-free controls from Jiangsu province

Variable	Cases (N=402)		Controls (N=473)		*P* [a]
	No.	%	No.	%	
Age range, month	0.033 - 168.00		0.367 - 168.00		
Mean ± SD	40.99 ± 35.49		40.88 ± 29.76		0.962 [b]
Age					0.100
≤18 months	139	34.58	139	29.39	
>18 months	263	65.42	334	70.61	
Sex					0.987
Female	191	47.51	225	47.57	
Male	211	52.49	248	52.43	
Site of origin					
Adrenal gland	93	23.13	/	/	
Retroperitoneal region	167	41.54	/	/	
Mediastinum	120	29.85	/	/	
Other region	18	4.48	/	/	
NA	4	1.00	/	/	
INSS stage					
I	108	26.87	/	/	
II	63	15.67	/	/	
III	59	14.68	/	/	
IV	104	25.87	/	/	
4s	2	0.50	/	/	
NA	66	16.42	/	/	

SD, standard deviation; NA, not available.

[a] Two-sided χ^2 test between neuroblastoma patients and cancer-free controls.

[b] t-test between neuroblastoma patients and cancer-free controls.
